# Cost‐effectiveness of couples’ voluntary HIV counselling and testing in six African countries: a modelling study guided by an HIV prevention cascade framework

**DOI:** 10.1002/jia2.25522

**Published:** 2020-06-30

**Authors:** Kristin M Wall, Mubiana Inambao, William Kilembe, Etienne Karita, Elwyn Chomba, Bellington Vwalika, Joseph Mulenga, Rachel Parker, Tyronza Sharkey, Amanda Tichacek, Eric Hunter, Robert Yohnka, Gordon Streeb, Phaedra S Corso, Susan Allen

**Affiliations:** ^1^ Rwanda Zambia HIV Research Group Department of Pathology & Laboratory Medicine School of Medicine and Hubert Department of Global Health Rollins School of Public Health Laney Graduate School Emory University Atlanta GA USA; ^2^ Department of Epidemiology Rollins School of Public Health Laney Graduate School Emory University Atlanta GA USA; ^3^ Department of Obstetrics and Gynecology Ndola Central Hospital Ndola Zambia; ^4^ Ministry of Health Lusaka Zambia; ^5^ Department of Obstetrics and Gynecology School of Medicine University of Zambia Lusaka Zambia; ^6^ Department of Pathology & Laboratory Medicine School of Medicine Emory University Atlanta GA USA; ^7^ Emory Vaccine Center Yerkes National Primate Research Center Emory University Atlanta GA USA; ^8^ Departments of Economics and Political Science Emory University Atlanta GA USA; ^9^ Office of Research Kennesaw State University Kennesaw GA USA

**Keywords:** Africa, cost‐effectiveness, costs and cost analysis, couples, HIV, HIV prevention cascade, prevention and control

## Abstract

**Introduction:**

Couples’ voluntary HIV counselling and testing (CVCT) is a high‐impact HIV prevention intervention in Rwanda and Zambia. Our objective was to model the cost‐per‐HIV infection averted by CVCT in six African countries guided by an HIV prevention cascade framework. The HIV prevention cascade as yet to be applied to evaluating CVCT effectiveness or cost‐effectiveness.

**Methods:**

We defined a priority population for CVCT in Africa as heterosexual adults in stable couples. Based on our previous experience nationalizing CVCT in Rwanda and scaling‐up CVCT in 73 clinics in Zambia, we estimated HIV prevention cascade domains of motivation for use, access and effectiveness of CVCT as model parameters. Costs‐per‐couple tested were also estimated based on our previous studies. We used these parameters as well as country‐specific inputs to model the impact of CVCT over a five‐year time horizon in a previously developed and tested deterministic compartmental model. We consider six countries across Africa with varied HIV epidemics (South Africa, Zimbabwe, Kenya, Tanzania, Ivory Coast and Sierra Leone). Outcomes of interest were the proportion of HIV infections averted by CVCT, nationwide CVCT implementation costs and costs‐per‐HIV infection averted by CVCT. We applied 3%/year discounting to costs and outcomes. Univariate and Monte Carlo multivariate sensitivity analyses were conducted.

**Results:**

We estimated that CVCT could avert between 54% (Sierra Leone) and 62% (South Africa) of adult HIV infections. Average costs‐per‐HIV infection averted were lowest in Zimbabwe ($550) and highest in South Africa ($1272). Nationwide implementations would cost between 7% (Kenya) and 21% (Ivory Coast) of a country’s President’s Emergency Plan for AIDS Relief (PEPFAR) budget over five years. In sensitivity analyses, model outputs were most sensitive to estimates of cost‐per‐couple tested; the proportion of adults in heterosexual couples and HIV prevention cascade domains of CVCT motivation and access.

**Conclusions:**

Our model indicates that nationalized CVCT could prevent over half of adult HIV infections for 7% to 21% of the modelled countries’ five‐year PEPFAR budgets. While other studies have indicated that CVCT motivation is high given locally relevant promotional and educational efforts, without required indicators, targets and dedicated budgets, access remains low.

## 1 INTRODUCTION

Incident HIV infections in sub‐Saharan Africa have fallen 13% over recent years due to global prevention efforts [1]. However, this decline in new infections is slowing, gaps in the scale‐up of treatment and prevention services persist, and flatlined funds are not projected to meet 2030 Sustainable Development Goals [1, 2]. Now more than ever, maximizing the value of limited funding is critical, and evaluating the cost‐effectiveness of HIV prevention and treatment strategies is essential for improved resource allocation [1, 3]. The HIV prevention cascade, which evaluates domains of intervention motivation for use, access and effectiveness among a priority population, provides a useful framework to evaluate and advocate for prevention interventions [4] and has been used to evaluate interventions such as pre‐exposure prophylaxis (PrEP), voluntary medical male circumcision (VMMC) and prevention of mother‐to‐child transmission (PMTCT) [5, 6, 7, 8].

The HIV prevention cascade has yet to be described or evaluated for couples’ voluntary HIV counselling and testing (CVCT), an evidence‐based intervention in which couples receive joint pre‐test counselling, testing and post‐test counselling with counsellor‐facilitated serostatus disclosure [9, 10]. CVCT decreases sexual and perinatal HIV incidence [11, 12, 13, 14, 15, 16] by educating and placing joint responsibility on the dyad to increase uptake of condoms, VMMC, family planning, antiretroviral therapy (ART) and PMTCT [13, 14, 15, 17]. The priority population for CVCT is stable couples. With the exception of South Africa, most African adults are in cohabiting sexual unions [18], and the majority of HIV transmissions in sub‐Saharan Africa occur in heterosexual HIV discordant or concordant HIV‐negative couples [19]. Despite World Health Organization (WHO) [9] and the US Centers for Disease Control and Prevention (CDC) [10] CVCT recommendations, only a small percentage of African adults have been tested with partners.

Building on our technical support for the nationalization of CVCT in antenatal clinics (ANC) in Rwanda, where >80% of couples are now tested [20], we recently reported the cost‐effectiveness of a CVCT demonstration project serving 207,428 Zambian couples in 73 government clinics [21]. Receiving CVCT was associated with a 79% reduction in seroincidence among discordant couples using ART, 63% among discordant couples not using ART and 47% among concordant‐negative couples. The cost‐per‐HIV infection averted (CHIA) for CVCT was $659. We then built and validated a deterministic compartmental model which incorporated key domains from the HIV prevention cascade framework and reported the CHIA for nationalizing CVCT in Zambia ($394 CHIA) [21].

In the present analysis, we adapt this model to estimate the proportion of adult HIV infections averted, total costs and CHIA for nationalizing CVCT in six countries across sub‐Saharan Africa.

## 2 METHODS

### 2.1 Ethics approval

This retrospective costing and modelling study used de‐identified data from the publicly available sources cited. The Institutional Research Board at Emory University waived informed consent requirements.

### 2.2 Deterministic compartmental model

Our CVCT CHIA model details are published [21]. In summary, a deterministic compartmental model with one‐year time steps based on a series of differential equations was developed in Excel which allows heterosexual adult couples who are either concordant HIV‐negative or discordant, and on ART or off ART, to transition between states of HIV status and/or ART use over time. Our previous model structure [21] has been adapted here to incorporate HIV prevention cascade domains of motivation and access, which the previous model did not include.

Our model uses HIV seroincidence rates in uncounselled (“pre‐CVCT”) serodiscordant and concordant‐negative couples (which is assumed to be a function of all current prevention programmes taking place in country) and applies the estimated effectiveness of CVCT among couples depending on their joint HIV serostatus and ART use. These effectiveness estimates are applied each year, and couples move from concordant negative to discordant and from discordant to concordant positive accordingly. Estimated “pre‐CVCT” ART use and ART initiation in the year following CVCT are also model parameters, with an additional proportion of HIV‐positive individuals taking up ART each year. Estimated costs‐per‐couple tested are based on our programmatic experience. The model outputs HIV infections averted by CVCT (by subtracting the cumulative infections that are projected to occur post‐CVCT from those occurring pre‐CVCT), total incremental financial costs to implement CVCT and incremental CHIA by CVCT [21]. As evidence suggests that the HIV prevention impact of CVCT is sustainable for at least five years [22, 23, 24, 25], we chose a five‐year time horizon.

**Figure 1:**
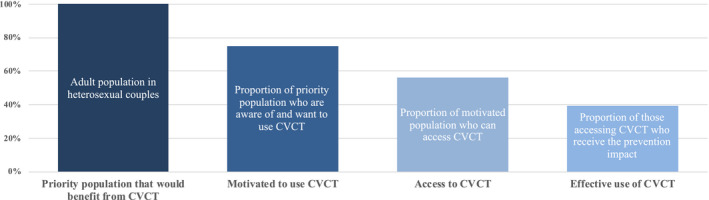
Generic HIV prevention cascade for CVCT. CVCT, couples’ voluntary HIV counselling and testing.

In the model, we conceptualize the expansion of CVCT in four phases: initiation, expansion, maturation and maintenance which are described in more detail below. These phases are defined by a changing set of estimated values for the HIV prevention cascade domains of motivation and access among the priority population for CVCT (Figure 1) and cost‐per‐couple tested.

Using this model, we previously estimated the cost‐effectiveness of nationalizing CVCT in Zambia by applying the actual financial expenditures and CVCT effectiveness observed when scaling‐up services in 73 government clinics in Lusaka, Copperbelt and Southern Provinces which reached 207,428 couples. We conducted sensitivity analyses to evaluate the effect of possible differential loss to follow‐up, informative censoring using inverse probability of censoring weighting [26, 27], and inverse probability of treatment weighting to evaluate the possibility of confounding when using observational data to estimate intervention effects. We found that our model was robust in these sensitivity analyses [21]. Additionally, to internally validate the model, we used logical testing by varying transition probabilities and setting costs and outcomes to 0 separately, which resulted in logical expected values.

### 2.3 Base‐case model parameters applied to all countries

Base‐case estimates for CVCT HIV prevention impact were derived from our CVCT implementation in 73 Zambian government clinics [21] (Table 1). Briefly, from September 2010 to March 2016, CVCT services were implemented including joint pre‐test counselling; rapid HIV testing; joint post‐test counselling; provision of condoms and referrals as needed for ART, VMMC and family planning. History of prior HIV testing and self‐reported ART use were documented. HIV antibody‐negative individuals had repeat tests one month after CVCT. Discordant couples returned for quarterly retesting and counselling, and concordant HIV‐negative couples returned for annual retesting and counselling [28]. Promotions utilized influential community health workers and mass media [28, 29].

**Table 1:** Base‐case model parameters applied to all countries

	Value and source
CVCT effective use (HIV prevention impact)	
Among concordant HIV‐negative couples	47% [21]
Among discordant couples not on ART	63% [21]
Among discordant couples on ART	79% [21]
ART use	
Immediate increase in uptake after CVCT	38% [21]
Additional uptake per year	5% [21]
CVCT motivation among the priority population	
Initiation phase	20% of couples [20, 21]
Expansion phase	38% of couples [20, 21]
Maturation phase	66% of couples [20, 21]
Maintenance phase	15% of couples [20, 21]
CVCT access among those motivated	
Initiation phase	50% of motivated couples [20, 21]
Expansion Phase	56% of motivated couples [20, 21]
Maturation phase	60% of motivated couples [20, 21]
Maintenance phase	70% of motivated couples [20, 21]

ART, antiretroviral treatment; CVCT, couples’ voluntary HIV counselling and testing; USD, United States Dollar.

During the implementation, the impact of CVCT was assessed over longitudinal follow‐up. HIV seroincidence rates for discordant and concordant HIV‐negative couples were calculated as incident infections divided by HIV‐negative person‐years (PY) of follow‐up, stratified by whether couples had (“post‐CVCT”) or had not (“pre‐CVCT”) received CVCT. As shown in Table 1, in concordant‐negative couples, we observed a 47% reduction in HIV seroincidence pre‐CVCT (1.1/100PY) versus post‐CVCT (0.6/100PY). In serodiscordant couples in which the HIV+ partner was not on ART, we observed a 63% reduction in incidence (13.0/100PY pre‐CVCT versus 4.8/100PY post‐CVCT). Finally, in serodiscordant couples in which the HIV+ partner was on ART, we observed a 79% reduction in incidence (8.5/100PY pre‐CVCT versus 1.8/100PY post‐CVCT) [21]. We also use the increase in ART uptake reported in the previously published manuscript (38% of non‐users initiated ART after CVCT with 5%/year additional uptake thereafter) [21]. Self‐reported ART initiation is not assumed to imply adherence/suppressive ART.

Base‐case estimates for cost‐per‐couple tested, motivation for CVCT, and access to CVCT were derived from both the Zambian implementation study described and our years of experience supporting nationalization of CVCT in Rwanda [20, 21]. We derived incremental financial costs from the donor’s perspective from a primary costing study of actual expenditures to implement CVCT in government clinics following Global Heath Cost Consortium guidance [30]. Cost data were reported by activity were recorded by study staff during programme implementation and entered in AccPac (Sage Group). Based on our experiences in Rwanda and Zambia [20, 21], motivation for CVCT, and access to CVCT varied over time and are used to define implementation phases as shown in Table 1: initiation phase (advocacy, training and promotions to motivate 20% of couples, 50% of whom have CVCT access); expansion phase (continued advocacy and training, increased community and politico‐administrative involvement, CVCT certification for a majority of providers and active promotions to motivate 38% of couples, 56% of whom have CVCT access); maturation phase (an established programme with CVCT integrated into existing services including ANC, individual HIV counselling and testing, ART, VMMC and family planning services and 66% of couples are motivated, 60% of whom have CVCT access); and finally the maintenance phase (for hard‐to‐reach residual and new couples where only 15% are motivated, of whom 70% have CVCT access). Thus, 80% of couples are assumed to be reached with testing, as seen in Rwandan ANC [20].

### 2.4 Country‐specific parameters

South Africa, Zimbabwe, Kenya, Tanzania, Ivory Coast and Sierra Leone have diverse HIV epidemics and published data available for the model inputs (Table 2). Country‐specific model parameters include: (1) proportion of adults in cohabiting heterosexual couples, (2) couple HIV serostatus distribution, (3) ART use, (4) HIV seroincidence in uncounselled (pre‐ CVCT) concordant‐negative couples, (5) HIV seroincidence in uncounselled discordant couples not on ART, (6) HIV seroincidence in uncounselled discordant couples on ART and (7) estimated cost‐per‐couple tested. Published estimates of HIV seroincidence in uncounselled concordant‐negative couples [31, 32, 33, 34] and uncounselled discordant non‐ART using couples [31, 32, 33, 34, 35] are limited to Eastern Africa. Given limited data for Southern African countries, model inputs 4, 5 and 6 (Table 2) were estimated from Zambia data [21]. As no published data are available for HIV seroincidence in uncounselled ART‐using discordant couples in Eastern and Western Africa, we used 5/100PY reflecting the broadly lower incidence in Eastern/Western versus Southern Africa [36]. Finally, since the primary cost driver for CVCT is salaries for counselling, testing and promotions [20, 21], we derived a conversion factor using country‐specific nurse salaries (USD 2015) applied to cost‐per‐couple tested estimates calculated from our Zambian implementation ($75 initiation phase, $50 expansion phase, $25 maturation phase, $30 maintenance phase [21]) to generate cost‐per‐couple tested estimates for each country.

**Table 2:** Country‐specific base‐case model parameters

Model input		Southern Africa								Eastern Africa								Western Africa						
		South Africa				Zimbabwe				Kenya				Tanzania				Ivory Coast				Sierra Leone		
		Value				Value				Value				Value				Value				Value		
1																								
Adult population (ages 15 to 64)	37,904,001		[37]		7 892 000		[37]		2 974 500		[37]		3 001 700		[37]		1 383 900		[37]		428 200		[37]	
Adult population in stable couples (%)	35%		[18]		58%		[18]		57%		[18]		57%		[18]		59%		[18]		62%		[18]	
2																								
Discordant couples among all stable couples (%)	16%		[18]		9%		[18]		6%		[18]		5%		[18]		5%		[18]		3%		[18]	
Concordant‐negative couples among all stable couples (%)	70%		[18]		80%		[18]		91%		[18]		91%		[18]		93%		[18]		97%		[18]	
3																								
Adults on ART of all estimated positive adults (%)	62%		[38]		89%		[38]		69%		[38]		72%		[38]		55%		[38]		43%		[38]	
4																								
Uncounselled seroincidence among concordant‐negative couples (per 100 PY)	1/100 PY		[21]		1/100 PY		[21]		0.5/100 PY		[31, 32, 33, 34]		0.5/100 PY		[31, 32, 33, 34]		0.5/100 PY		^a^		0.5/100 PY		^a^	
5																								
Uncounselled seroincidence among non‐ART using discordant couples (per 100 PY)	13/100 PY		[21]		13/100 PY		[21]		10/100 PY		[31, 32, 33, 34, 35, 39]		10/100 PY		[31, 32, 33, 34, 35, 39]		10/100 PY		^a^		10/100 PY		^a^	
6																								
Uncounselled seroincidence among ART using discordant couples (per 100 PY)	8/100 PY		[21]		8/100 PY		[21]		5/100 PY		^a^		5/100 PY		^a^		5/100 PY		^a^		5/100 PY		^a^	
7																								
Cost‐per‐couple tested (2015 USD)			[21, 40]				[21, 40]				[21, 40]				[21, 40]				[21, 40]				[21, 40]	
Initiation phase	$229				$84				$59				$73				$103				$71			
Expansion phase	$153				$56				$39				$49				$69				$47			
Maturation phase	$76				$28				$20				$24				$34				$24			
Maintenance phase	$92				$34				$24				$29				$41				$28			

“Uncounselled” indicates pre‐couples’ voluntary HIV counselling and testing. Demographic Health Survey defines stable couples as partners consensually living together in a union within a household. Most recent Demographic Health Survey data used. ART, antiretroviral treatment; PY, person year.

a Estimated based on lower HIV incidence in Eastern and Western Africa versus Southern Africa [36].

### 2.5 Base‐case analyses

The estimated proportion of adult HIV infections averted, total CVCT implementation costs, CHIA and proportion of President’s Emergency Plan for AIDS Relief (PEPFAR) budgets required for nationwide implementation in each selected country are outcomes of interest. These are presented alongside per capita gross domestic products (GDPs) for context. All outcomes and costs were discounted at 3%/year as recommended by the US Public Health Service Task Force [41]. We adhered to Consolidated Health Economic Evaluation Reporting Standards [42] for cost‐effectiveness analyses.

### 2.6 One‐way and probabilistic sensitivity analyses

We conducted one‐way sensitivity analyses for all model inputs by varying each parameter ±20%. Inputs which most influenced model outputs are reported. Because key model parameters of cost‐per‐couple tested and CVCT effectiveness were derived from just two countries, we also conducted probabilistic Monte Carlo simulation multivariate sensitivity analyses for each parameter of interest (±50% of base‐case estimates using a uniform distribution) with 1000 draws in Excel. Average outcomes and standard deviations from simulated results are reported. A uniform distribution was chosen to fix a functional form on the parameter estimates and to reflect a large degree of uncertainty around the selected parameters.

## 3 RESULTS

The total cost for nationwide CVCT implementation and cumulative infections averted are also shown in Table 3. Estimated average CHIA ranged from $1272 in South Africa to $550 in Zimbabwe. Our model estimated that CVCT could prevent between 54% and 62% of HIV infections. The proportion of the 2018 PEPFAR budget required for CVCT nationalization over five years ranged from 7% in Kenya to 21% in Ivory Coast. For context, per capita GDP for each country is shown.

**Table 3:** Proportion of adult infections averted, overall CHIA and total cost for CVCT in six African countries (primary base‐case analyses)

Southern Africa	Total cost of CVCT	Cumulative HIV infections averted	Average CHIA^a^	Proportion of infections averted, %	2018 PEPFAR Budget [43]	Cost of CVCT as % of PEPFAR budget for five years, %	Per capita GDP
South Africa	$532,704,861	418,855	$1272	62	$575,258,390	19	$13,054
Zimbabwe	$67,053,208	121,984	$550	58	$145,546,200	9	$2,224
East‐Central Africa							
Kenya	$176,419,535	231,312	$763	57	$505,480,000	7	$3,384
Tanzania	$219,582,392	219,486	$1000	56	$512,422,250	9	$3,094
Western Africa							
Ivory Coast	$145,955,594	119,508	$1221	57	$140,508,601	21	$3,771
Sierra Leone	$33,113,126	34,803	$951	54	Unknown	–	$1,547

No PEPFAR budget reported for Sierra Leone. Per capita GDP (2017 estimates in 2015 USD): https://www.cia.gov/library/publications/the‐world‐factbook/rankorder/2004rank.html. CHIA, cost per HIV infection averted; CVCT, Couples' HIV voluntary counselling and testing; PEPFAR: President's Emergency Plan for AIDS Relief.

a Weighted average across all implementation phases.

**Figure 2:**
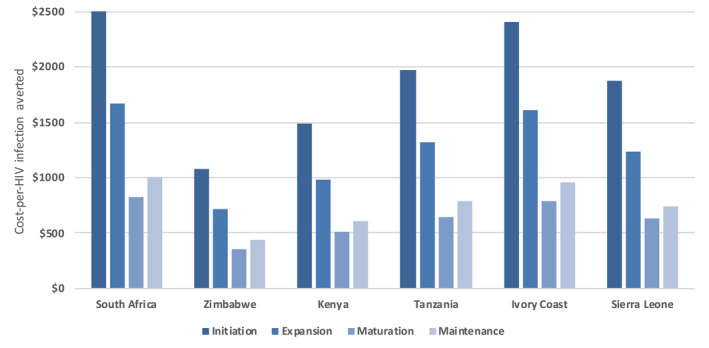
Modelled cost‐per‐HIV infection averted by phase of CVCT implementation in six sub‐Saharan African countries. CVCT, couples' HIV voluntary counselling and testing.

Figure 2 presents country‐specific CHIA estimates by implementation phase. During the initiation phase, the CHIA ranged from $2503 in South Africa to $1080 in Zimbabwe. During the expansion phase, the CHIA ranged from $720 to $1672. During the maturation phase, the CHIA ranged from $360 to $831. Finally, during the maintenance phase, the CHIA ranged from $437 to $1005.

### 3.1 Sensitivity analyses

One‐way sensitivity (Table 4 presents representative findings from South Africa) analyses indicated our model was relatively robust to parameter assumptions, with resulting CHIA still relatively low compared to other HIV prevention interventions (discussed below). Our model was most sensitive to the costs‐per‐couple tested (varying input parameters by ±20% resulted in total CVCT costs which were ±20% different from base‐case). Our model was also sensitive to varying the proportion of adults in heterosexual couples (a higher proportion of adults in heterosexual couples lead to a proportional increase in HIV infections averted and total CVCT costs). Similarly, our model was sensitive to the proportion of couples motivated for and with access to CVCT (with increasing motivation and access was associated with proportional changes in increased infections averted and costs).

**Table 4:** Parameters and results of one‐way sensitivity analyses: Illustrative example of South Africa

Sensitivity analysis parameters	Infections averted % change^a^	Total cost of CVCT % change^a^	CHIA % change^a^
Discount rate			
2%	3%	5%	2%
4%	−3%	−5%	−2%
Cost/couple tested (by implementation phase)			
$183; $122; $61; $74	0%	−20%	−20%
$274; $184; $91; $110	0%	20%	20%
Proportion adults in heterosexual couples			
28%	−20%	−20%	0%
42%	20%	20%	0%
Proportion couples motivated for CVCT (by implementation phase)			
40%; 40%; 53%; 12%	−20%	−20%	0%
60%; 60%; 79%; 18%	20%	20%	0%
Proportion couples with access to CVCT (by implementation phase)			
16%; 32%; 48%; 56%	−20%	−20%	0%
24%; 48%; 72%; 84%	20%	20%	0%
Proportion concordant negative			
56%	−9%	0%	10%
84%	10%	0%	−9%
Proportion discordant			
13%	−9%	0%	10%
19%	11%	0%	−10%
HIV seroincidence rates before CVCT (per 100 PY)			
Among concordant HIV‐negative couples			
0.80	−9%	0%	10%
1.20	9%	0%	−8%
Among ART using HIV discordant couples			
6.40	−6%	0%	6%
9.60	6%	0%	−6%
Among non‐ ART using HIV discordant couples			
10.40	−4%	0%	4%
15.60	4%	0%	−4%
CVCT prevention impact			
Among concordant HIV‐negative couples			
38%	−7%	0%	8%
56%	7%	0%	−7%
Among ART using HIV discordant couples			
63%	−10%	0%	11%
95%	11%	0%	−10%
Among non‐ART using HIV discordant couples			
50%	−2%	0%	2%
76%	2%	0%	−2%
ART use			
Among HIV‐positive adults before CVCT			
50%	2%	0%	−2%
74%	−3%	0%	3%
Among HIV‐positive adults after CVCT			
61%	−1%	0%	1%
92%	1%	0%	−1%
Proportion initiating ART each year after CVCT			
4%	0%	0%	0%
6%	0%	0%	0%

ART, antiretroviral treatment; CHIA, cost‐per‐HIV infection averted; CVCT, couples’ voluntary HIV counselling and testing; PY, person‐years.

a % Change relative to base‐case primary analyses.

Additionally, our model was relatively sensitive (with results varying roughly ±10% relative to base‐case) to the proportion of concordant negative and discordant couples in the population (with higher proportions of at‐risk couples leading to increased infections averted and therefore lower CHIAs).

A higher pre‐CVCT seroincidence rate in concordant‐negative couples and higher CVCT effectiveness in concordant‐negative couples both increased HIV infections averted. Finally, increasing CVCT effectiveness in ART using discordant couples by ±20% varied total infections averted by roughly ±10% (with higher prevention impact leading to increased infections averted and improved cost‐effectiveness).

Examining the country‐specific parameters specifically, differences in model outcomes across countries are influenced by differences in seroincidence rates pre‐CVCT (with higher uncounselled seroincidence rates being associated with higher numbers of infections averted and improved cost‐effectiveness) and the proportion of couples who were discordant (with higher discordancy associated with higher numbers of infections averted and improved cost‐effectiveness).

Probabilistic multivariate sensitivity analyses (Table 5) also highlighted that total CVCT costs were sensitive to cost‐per‐couple tested, with coefficients of variation of 15% to 16%. Our model was less susceptible to variation in CVCT impact, with coefficients of variation of 10% to 12%.

**Table 5:** Probabilistic sensitivity analysis results

Varying costs‐per‐couple tested by ±50% (uniform distribution) of the base‐case estimates^a^						
Southern Africa	Total cost of CVCT	SD	CV	Average CHIA	SD	CV
South Africa	527,493,591	80,271,276	15%	$1280	$198	15%
Zimbabwe	67,478,299	9,792,498	15%	$466	$73	16%
East‐Central Africa						
Kenya	175,560,051	28,074,065	16%	$765	$116	15%
Tanzania	219,184,615	33,759,017	15%	$997	$158	16%
Western Africa						
Ivory Coast	145,820,457	22,312,561	15%	$1217	$191	16%
Sierra Leone	33,384,147	4,850,786	15%	$953	$145	15%

Varying CVCT impacts by ±50% (uniform distribution) of the base‐case estimates^b^						
Southern Africa	Cumulative HIV infections averted	SD	CV	Average CHIA	SD	CV
South Africa	420,653	45,818	11%	$1304	$146	11%
Zimbabwe	116,862	14,257	12%	$570	$68	12%
East‐Central Africa						
Kenya	232,821	25,860	11%	$788	$86	11%
Tanzania	220,013	25,895	12%	$1043	$120	12%
Western Africa						
Ivory Coast	118,752	12,769	11%	$1269	$131	10%
Sierra Leone	34,876	4091	12%	$998	$120	12%

CHIA, cost per HIV infection averted; CV, coefficient of variation is the standard deviation divided by the mean estimate; CVCT, couples voluntary HIV counselling and testing; SD, standard deviation.

a No impact on cumulative HIV infections averted;

b no impact on total cost of CVCT.

## 4 DISCUSSION

Our model estimated that CVCT could prevent over half of adult HIV infections for 7% to 21% of selected countries’ five‐year PEPFAR budgets. Applying an HIV prevention cascade framework [5] is helpful to evaluate CVCT. In the countries under study, the priority population for CVCT, stable couples, comprises 35% to 62% of the adult population. Key barriers and solutions related to this priority population’s motivation to use CVCT, CVCT access and effective use are summarized in Table 6 and discussed in detail below.

**Table 6:** CVCT HIV prevention cascade domains with key barriers and solutions

Motivation	Access	Effective use
Reasons for gap		
Lack of knowledge and low risk perception	Lack of availability or easy access in government facilities	Inconsistent condom use, continued outside partner risk Lack of ART uptake
Lack of men's ability to attend regular clinic hours, opportunity costs	Lack of trained government providers	
Concerns about CVCT consequences	Perceived cost/affordability	Non‐linkage to ART programmes
Evidence‐based ways to close the gap		
Interventions		
Incentives/transport reimbursement Partner tracing, male‐focused sessions, 'expert couple' and influential community leader promotions Informational messaging highlighting partner safety, U = U, and addressing common concerns	Convenient service delivery hours and platforms Provider training and reimbursement (possibly during off‐hours)	Ongoing condom and behavioural counselling, targeted safe conception and alcohol counselling Integration with ART (for treatment and prevention) programmes
Platforms to deliver interventions		
Clinics, community health workers, influential peers and mass media	Clinic‐based services, mobile testing, home‐based testing, self‐testing	Clinic‐based services, mobile testing, home‐based testing, self‐testing
Policies to support interventions		
Budgets for training messaging, demand creation and incentives	Budgets, required reporting indicators and targets for CVCT	Budgets for integrated services, ongoing M&E
	Training and reimbursement of providers	

ART, antiretroviral treatment; CVCT, couples voluntary HIV counselling and testing; M&E, monitoring and evaluation.

### 4.1 Motivation

Sensitivity analyses indicated countries with a higher proportion of heterosexual couples motivated to uptake CVCT prevented more infections. Motivation for CVCT has been high across studies in diverse populations including heterosexual couples in Mozambique [44, 45], Tanzania [46, 47], South Africa [48, 49], Uganda [50], Thailand [51], Iran [52] and men who have sex with men (MSM) in South Africa [53] and the US [54, 55]. Although promising, these are relatively small‐scale efforts: knowledge of CVCT remains low in many settings and education and demand creation are essential to increase broad motivation [28, 29, 56]. Key barriers to motivation include lack of community knowledge of HIV serodiscordance and HIV risk. For example we found that only 30% of couples seeking CVCT in Durban knew about serodiscordance [49]. Other barriers to motivation include opportunity costs, men’s inability to attend regular clinic hours, limited knowledge that CVCT services exist, and concerns about consequences like relationship dissolution [28, 29, 56, 57].

Reports highlight successful strategies to overcome these barriers and increase motivation including use transport reimbursement [57]; nonmonetary incentives in Zambia [58] and rural Zimbabwe [59]; invitations plus facilitated contact tracing to support partner attendance in Malawi [60]; male‐focused interactive sessions with testimonies from ‘expert couples’ who received CVCT in rural Uganda [50]; and CVCT invitations and promotions delivered by influential community leaders and via mass media in Zambia and Rwanda [28, 29, 56]. Additionally, the desire to keep one’s partners safe from transmission is a motivating factor for ART and PrEP use [61, 62], and “undetectable = untransmittable” messaging may reduce stigma and motivate couples to uptake CVCT and ART to achieve viral suppression [63]. Finally, messaging should emphasize that outcomes of intimate partner violence (IPV), relationship dissolution or emotional distress are rare and CVCT typically *strengthens* relationships [16, 17, 54, 60, 64, 65]. To broadly increase CVCT motivation, budgets for training, demand creation and incentives (all included in our modelled CVCT costs) will be required.

### 4.2 Access

Sensitivity analyses indicated that countries with a higher proportion of heterosexual couples with CVCT access will prevent more infections at a higher cost. The PEPFAR 2020 Country Operation Plan [43] highlights family HIV testing and emphasizes that HIV prevention among pregnant, postpartum and breastfeeding women should include “couples‐based services to promote scaled‐up testing and treatment of male partners.” However, as CVCT is not broadly offered as standard of care in the selected countries, nor it is a required indicator for reporting [66], access remains low across Africa. As with most interventions, a main barrier to access is the cost of wide‐spread implementation. As expected, higher costs‐per‐couple tested increase total CVCT costs. Economies of scale are incorporated into estimated cost‐per‐couple tested (over time, costs to test couples decrease, with the exception of the final “hard‐to‐reach” couples).

Our experience in Zambia reflected the initiation and expansion phases [21]. Government clinic staff were paid during their off‐duty hours. Unfortunately, the amount of funding available was insufficient to maintain momentum. In contrast, Rwanda succeeded in increasing access and nationalizing CVCT in ANC between 2008 and 2013 (after which CVCT become a social norm and demand creation was no longer needed) [20]. Based on the estimated impact of CVCT in Rwanda from observational epidemiological studies [11, 23, 24], the Rwandan government health insurance and performance‐based financing plans now reimburse the costs of CVCT, and additional funding for off‐duty staff is no longer required. Reaching Rwanda’s success will require investment. As many demonstration projects correspond to the more expensive initiation phase, implementers may fail to see that continuous investment is necessary to achieve social diffusion, incorporate CVCT into daily clinical practice and adapt data recording and reporting tools to achieve sustained access [57].

In addition to clinic‐based CVCT, mobile testing in Rwanda [29]; home‐based couples’ testing in Tanzania [67], Kenya [68] and Malawi [69]; and self‐testing to increase CVCT in Kenya [70] have been studied. An improved understanding of differences in CVCT access and costs (as well as motivation) for different modalities is warranted.

### 4.3 Effective use

CVCT decreases sexual and perinatal HIV incidence [11, 12, 13, 14, 15, 16] by educating and placing joint responsibility on the dyad to increase uptake of condoms, VMMC, family planning, ART and PMTCT [13, 14, 15, 17]. CVCT effectiveness must be monitored to understand facilitators and barriers to achieving reductions in HIV incidence. Our previous analyses found factors associated with inconsistent condom use, non‐ART initiation or continued outside partner risk in discordant couples post‐CVCT included alcohol use and fertility intentions [71]. To improve effective use of CVCT for all couples, targeted safe conception or alcohol counselling may be warranted. Importantly, as seen in sensitivity analyses, a higher prevention impact in concordant‐negative couples increased CVCT cost‐effectiveness. It has been argued that CVCT may have a sizable impact on the epidemic through HIV prevention in concordant‐negative couples (via reduction in outside relationship risk) since they comprise the majority of the population [72, 73].

For the smaller though higher‐risk population of discordant couples, “Test‐and‐Treat” will continue to expand. Unfortunately, many country’s HIV incidence rates have not decreased as substantially as predicted in the test‐and‐treat era, and several large cluster‐randomized trials have failed to clearly demonstrate the population‐level prevention impact of universal test‐and‐treat policies [74]. Where CVCT increases ART uptake and adherence in serodiscordant couples, it may bolster the effectiveness of test‐and‐treat. Additionally, to achieve the PEPFAR 2020 priority of PrEP for discordant couples [43], CVCT can effectively identify discordant couple candidates. Although PrEP is not currently available in most countries outside of relatively small demonstration and research projects, CVCT counselling can be updated to include PrEP as it becomes available.

Finally, an improved understanding of the effectiveness of CVCT in home‐based settings, mobile‐testing and via self‐testing is needed with regular monitoring and evaluation of these delivery platforms. With self‐testing in particular, how well couples disclose, understand their respective results and adopt appropriate risk reduction without facilitated joint post‐test counselling merits further investigation [75, 76, 77, 78].

### 4.4 Comparative cost‐effectiveness

We found CVCT CHIA estimates to be similar to interventions largely considered cost‐effective including individual voluntary HIV counselling and testing (estimated in a previous systematic review of studies in sub‐Saharan Africa at $1315 [79] and $483 for an individual and couples testing intervention in Kenya [79, 80]) and family planning for PMTCT via prevention of unintended pregnancy ($663) [79]. A recent systematic review of 60 studies from African countries reported median CHIA estimates for VMMC ($2965), ART for PMTCT ($1144), treatment‐as‐prevention interventions ($7903) and PrEP ($13,267) [81].

### 4.5 Limitations

As in all models, we attempt to simplify a complex reality, and outputs are dependent on assumptions. Extensive sensitivity analyses quantify the impact of these assumptions. Our model seeks to isolate the impact of CVCT on HIV infections averted given constant pre‐CVCT and post‐CVCT HIV incidence. We do not attempt to predict the course of the epidemic in the selected countries over time by considering myriad other prevention or treatment interventions. More detailed models isolating the prevention impact of CVCT attributable to condom use, VMMC uptake, improved ART uptake and adherence, and/or reductions in concurrent relationships are warranted. While deterministic compartmental models are well‐suited to examine average characteristics in a population and are thus appropriate for our goal, they do not evaluate individual‐level effects as do agent‐based models. Finally, while some studies of HIV prevention interventions translate infections averted into disability‐adjusted life‐years averted or quality‐adjusted life‐years gained, a recent systematic review did not find standard conversions applicable across country settings [81]. While such cost‐utility estimates are often applied to determine if an intervention is cost‐effective [82], this threshold is often questioned by experts since it does not consider intervention affordability [83, 84]. While cost‐utility analyses are useful for comparing interventions with different natural units, given dedicated HIV prevention budgets and the common use of CHIA estimates in other studies [81], we feel that CHIA is a more useful comparative measure.

## 5 CONCLUSIONS

Our model indicated that nationalized CVCT could prevent over half of adult HIV infections for 7% to 21% of the modelled countries’ five‐year PEPFAR budgets. Unfortunately, WHO CVCT guidelines have yet to be broadly implemented. While studies indicate that CVCT motivation is high given locally relevant promotional and educational efforts, access remains low without dedicated budgets or required indicators.

## COMPETING INTEREST

The authors have no conflicts of interest.

## AUTHORS’ CONTRIBUTIONS

KMW contributed to the analysis and interpretation of data; drafted the article and revised it critically for important intellectual content; and gave final approval of the version to be published. MI contributed to the conception and design of the study, revised the article critically for important intellectual content and gave final approval of the version to be published. WK contributed to the conception and design of the study, revised the article critically for important intellectual content and gave final approval of the version to be published. EK contributed to the conception and design of the study, revised the article critically for important intellectual content and gave final approval of the version to be published. EC contributed to the conception and design of the study, revised the article critically for important intellectual content and gave final approval of the version to be published. BV contributed to the conception and design of the study, revised the article critically for important intellectual content and gave final approval of the version to be published. JM contributed to the conception and design of the study, revised the article critically for important intellectual content and gave final approval of the version to be published. RP contributed to the analysis and interpretation of data; revised the article critically for important intellectual content and gave final approval of the version to be published. TS contributed to the analysis and interpretation of data; revised the article critically for important intellectual content and gave final approval of the version to be published. AT contributed to the study conception and design, revised the article critically for important intellectual content and gave final approval of the version to be published. EH contributed to the analysis and interpretation of data; revised the article critically for important intellectual content and gave final approval of the version to be published. RY contributed to the analysis and interpretation of data; revised the article critically for important intellectual content and gave final approval of the version to be published. GS contributed to the analysis and interpretation of data; revised the article critically for important intellectual content and gave final approval of the version to be published. PC contributed to the analysis and interpretation of data; revised the article critically for important intellectual content and gave final approval of the version to be published. SA contributed to the study design and conception, contributed to the analysis and interpretation of data; revised the article critically for important intellectual content and gave final approval of the version to be published.
